# Effect of a luteal phase rescue protocol on live birth rates in frozen embryo transfer cycles

**DOI:** 10.3389/frph.2025.1547939

**Published:** 2025-09-30

**Authors:** Chadi Yazbeck, François Olivennes, Nadia Kazdar, Claire Pietin-Vialle, Solenne Gricourt

**Affiliations:** ^1^ Ambroise Paré—Cherest ART, Centre for Reproductive Medicine, Groupe Hospitalier Privé Ambroise Paré Hartmann, Neuilly Sur Seine, France; ^2^ Obstetrics Gynecology and Reproductive Medicine, Reprogynes Medical Institute, Paris, France; ^3^ Department of Reproductive Medicine, Bichat Claude Bernard Hospital, Assistance Publique - Hôpitaux de Paris (APHP), Paris, France

**Keywords:** luteal phase support, frozen embryo transfer, progesterone, hormone replacement therapy, endometrial receptivity, live birth rate

## Abstract

**Introduction:**

Frozen embryo transfer (FET) is a standard procedure that improves live birth rates and reduces ovarian hyperstimulation risks. Optimizing luteal phase support with hormone replacement therapy (HRT), particularly by progesterone supplementation, enhances endometrial receptivity and embryo implantation success. Despite advances in cryopreservation techniques, optimal protocols for progesterone supplementation in HRT-FET cycles remain uncertain. This study aims to evaluate the effects of an individualized luteal phase protocol using subcutaneous progesterone on live birth rates in HRT-FET cycles.

**Methods:**

In this retrospective cohort study, we analyzed data from 433 autologous FET cycles prepared with HRT. Serum progesterone levels were measured the day before FET. Two groups were compared according to serum progesterone measurement the day before FET. The control group (≥ 11 ng/ml), received standard luteal support (800 mg vaginal progesterone daily); and the rescue group (<11 ng/ml), received an additional 25 mg subcutaneous progesterone daily. Pregnancy outcomes, including biochemical pregnancy, clinical pregnancy, miscarriage, and live birth rates, were assessed across both groups.

**Results:**

Despite overall similar pregnancy rates, the rescue group, receiving combined subcutaneous and vaginal progesterone, demonstrated a higher live birth rate compared to the control group (36.9% vs. 24.7%, *p* = 0.006). By Day 12 after FET, progesterone levels in the rescue group were comparable to those in the control group.

**Conclusion:**

Our findings suggest that adding subcutaneous progesterone to standard vaginal support in HRT-FET cycles may improve reproductive outcomes in patients with low serum progesterone levels the day before transfer. These results support tailoring progesterone supplementation to optimize luteal phase support. Further controlled trials are needed to establish standardized protocols for HRT-FET cycles.

## 1 Introduction

Frozen embryo transfer (FET) has become an increasingly preferred practice in assisted reproductive technology (ART), offering several advantages over fresh embryo transfers, including a reduced risk of ovarian hyperstimulation syndrome (1). In the early years of *in vitro* fertilization (IVF), FET was limited by low embryo survival after thawing (2). Advances in cryopreservation techniques have since improved embryo viability and markedly increased pregnancy success rates. In the 1980s, FET success rates were below 15%, but by the early 2000s live birth rates had risen to approximately 27% per transfer, making FET a viable and often favorable option (2, 3). The success of FET depends on adequate endometrial preparation to ensure optimal receptivity, typically achieved with hormone replacement therapy (HRT). Estrogen is administered to promote endometrial growth, followed by progesterone to induce secretory changes required for a receptive environment. The timing of progesterone exposure before embryo transfer is crucial for endometrial receptivity (4). Some studies suggest that the optimal duration of progesterone exposure should align with the embryo's developmental stage plus one additional day—six days for blastocysts and four days for cleavage-stage embryos (5). Randomized controlled trials indicate that shorter progesterone exposure may be associated with increased early pregnancy loss, but show no significant differences in live birth rates across varying durations of progesterone therapy (6, 7). A cohort study also reported that extending progesterone exposure to seven days for blastocysts was associated with lower live birth rates than six days (8). Evidence regarding exogenous progesterone favors individualized luteal phase support to optimize pregnancy outcomes, and specific cutoff levels have been proposed since 2017 (9). Despite various FET regimens, a Cochrane meta-analysis found insufficient evidence to recommend a single approach for subfertile women with regular ovulatory cycles, leaving clinical practice to guide regimen choice (10). In HRT-FET cycles, proper timing of embryo transfer remains crucial; limited evidence suggests that Day 5/6 blastocysts benefit from transfer on the fifth or sixth day of progesterone exposure, while cleavage-stage embryos may be optimally transferred on the third or fourth day (11). A key challenge in HRT-FET cycles is maintaining optimal serum progesterone levels, as inadequate levels can compromise implantation and live birth rates. Factors such as pharmacokinetics, body mass index (BMI), age, and other individual characteristics contribute to variability in serum progesterone (12, 13). Recent studies have shown improved pregnancy outcomes when serum progesterone exceeds thresholds ranging from 8.75 to 32.5 ng/ml, while levels below 10.64 ng/ml are linked to significant reductions in live birth rates (14–16). Receiver operating characteristic (ROC) analyses have established an optimal progesterone threshold for ongoing pregnancy, with acceptable sensitivity and specificity between 10.7 and 12.3 ng/ml (9, 17). To address inadequate luteal support, recent studies have introduced “rescue” protocols that typically supplement standard vaginal progesterone with subcutaneous injections, providing a reliable means of increasing serum progesterone levels for patients with insufficient initial values. This approach has been associated with improved implantation and pregnancy outcomes (18, 19).

Nevertheless, little is known about the optimal type, dose and timing of additional progesterone supplementation in luteal phase and early pregnancy. To date, limited evidence confirms whether an additional supplementation of progesterone improves live birth outcomes or requires adjustments based on administration route. This study aims to evaluate the effect of a luteal phase rescue protocol using subcutaneous progesterone on live birth rates in HRT-FET cycles, contributing to an evidence-based approach for individualized luteal phase support in ART.

## 2 Material and methods

### 2.1 Study design and population

**Figure 1:**
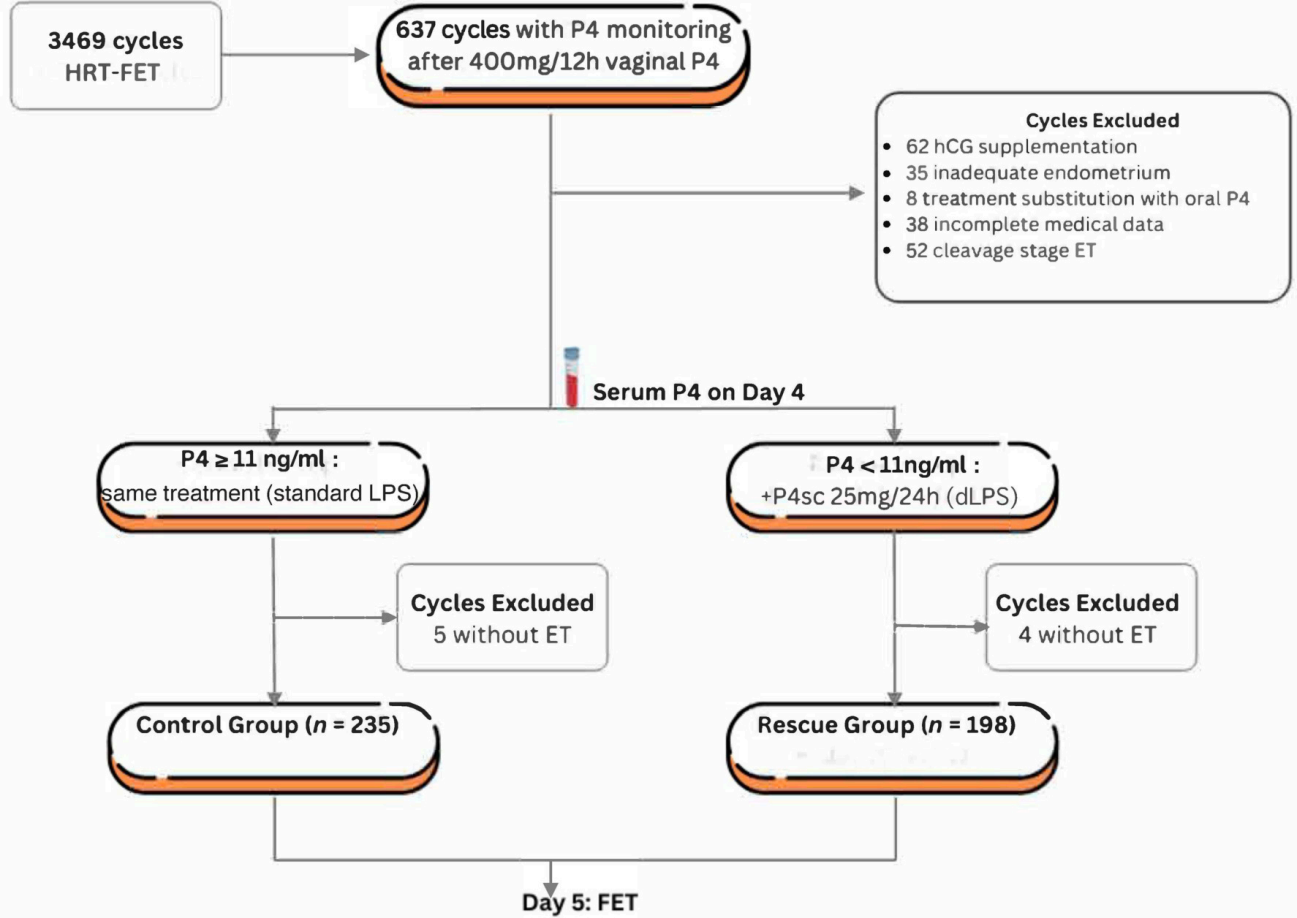
Flow diagram of patients’ selection*.* Flow diagram showing patient distribution into groups according to serum progesterone levels on the day before frozen embryo transfer. From the initial 3,469 HRT cycles with FET, 637 cycles were HRT with luteal phase support of 800 mg/12 h of progesterone until FET. Of these, 62 cycles were excluded due to HCG dose administration (add-on treatment), 52 cycles due to Day 2/Day 3 embryo transfer, 38 cycles because of missing or incomplete data, 35 cycles for inadequate endometrial preparation, and eight cycles due to treatment substitution. HRT, Hormone Replacement Therapy; P4, progesterone; FET, frozen embryo transfer; d-LPS, double route luteal phase support.

This retrospective study was conducted at Ambroise Paré—Cherest Center for Reproductive Medicine in Neuilly, France, between January 2019 and September 2022, in infertile couples undergoing IVF cycles with HRT for endometrial preparation and subsequent FET. Inclusion criteria were patient age at oocyte retrieval between 18 and 43 years, undergoing FET after failed fresh ET, after a ‘freeze all’ IVF cycle, or after a successful delivery (returning for a second child). Only single or double blastocyst-stage embryo transfers were included. All HRT cycles in this study cohort received individualized luteal phase support, followed by autologous frozen blastocyst transfer without PGT-A assessment. Of the initial 3,469 HRT-FET cycles, 637 cycles monitored by three clinicians were systematically evaluated with progesterone measurement the day before transfer (Day 4 after luteal phase support of 400 mg progesterone every 12 h. Of these, 62 cycles were excluded due to HCG administration for implantation issues, 52 cycles because of cleavage-stage transfers, 38 cycles for missing or incomplete data, and eight cycles due to treatment substitution with oral progesterone. In addition, 35 cycles were canceled for inadequate endometrial preparation (Figure 1). The final cohort consisted of 433 cycles involving 254 patients. Data were obtained from medical records and entered into a database, with secondary verification as needed. Extracted data were anonymized and assigned unique identifiers. All patients provided informed consent for the use of their medical data in accordance with the local institutional review board policy.

### 2.2 Study protocol and group setting

#### 2.2.1 Embryo culture

All fertilized oocytes were cultured in an incubator (G210 Incubator, K-Systems, CooperSurgical®) using single-step culture media (SAGE 1-Step™, CooperSurgical®). Embryo quality and grading were determined by morphology and development criteria (Gardner Scale) (20). High-quality Day 5/6 embryos were vitrified using the Vit Kit—Freeze (FUJIFILM Irvine Scientific) and transferred in a subsequent cycle.

#### 2.2.2 Endometrial preparation and group allocation

**Figure 2:**
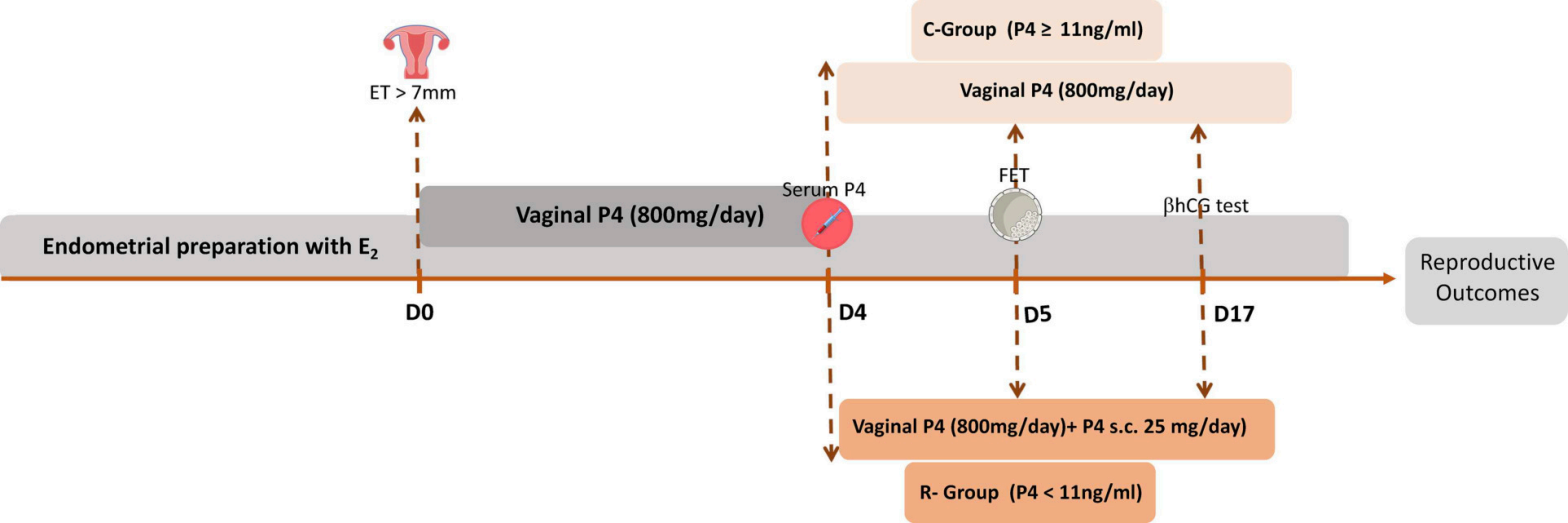
Endometrial preparation and patient stratification*.* Endometrial preparation followed a standard hormone replacement therapy protocol, beginning with oral estradiol at 4 mg daily, increased to 6 mg daily after day 9. Once endometrial thickness exceeded 7 mm, 400 mg (twice daily) of vaginal progesterone was administrated. Serum progesterone levels were measured one day before frozen embryo transfer (FET) to guide individualized luteal phase support. Patients were divided into two groups based on serum progesterone levels. The R-Group (*n* = 198) included patients with low progesterone levels (<11 ng/ml) who received 800 mg of vaginal progesterone and 25 mg of subcutaneous progesterone every 24 h. The C-Group (*n* = 235) consisted of patients with normal progesterone levels (≥11 ng/ml) who received 800 mg of vaginal progesterone alone. ET, endometrial thickness; FET, frozen embryo transfer; P4, progesterone.

Endometrial preparation followed a standard HRT fixed protocol, starting with oral estradiol at 4 mg daily from Day 1 and increasing to 6 mg daily after Day 9. When endometrial double-layer thickness exceeded 7 mm, vaginal progesterone was initiated at 400 mg twice daily. Serum progesterone levels were measured one day before FET to guide luteal support. Patients were stratified into two groups: Rescue Group (R-Group; *n* = 198): patients with low serum progesterone (<11 ng/ml) received both 400 mg vaginal progesterone every 12 h and 25 mg of subcutaneous progesterone (Progiron® IBSA, France) every 24 h. Control Group (C-Group; *n* = 235): patients with normal serum progesterone (≥11 ng/ml) received 400 mg vaginal progesterone alone every 12 h (Figure 2).

#### 2.2.3 Progesterone measurement

**Figure 3:**
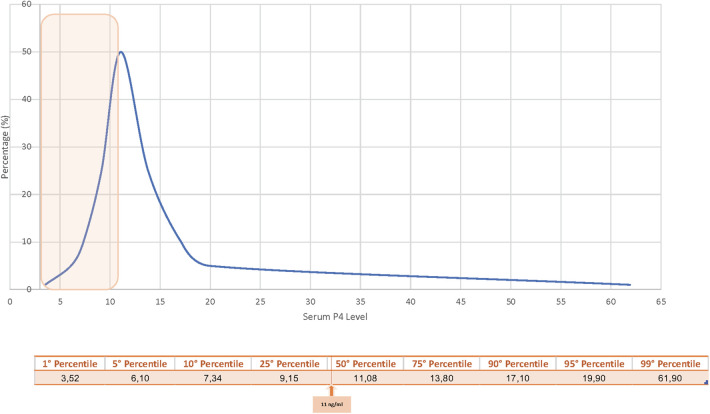
Serum progesterone distribution at Day 4. Serum progesterone levels on day 4 of vaginal progesterone administration were assessed using the Abbott Architect Progesterone assay. Patients below the pre-determined threshold of 11 ng/ml (orange square) received additional subcutaneous progesterone supplementation to optimize hormonal support before FET. The 50th percentile of progesterone level observed in the cohort was 11.08 ng/ml.

Serum progesterone was assessed using the Abbott Architect Progesterone assay, a high-sensitivity system with a detection limit of <0.1 ng/ml and coefficients of variation of 6.9% (low) and 4.6% (high). Measurements were performed in the same laboratory (Eylau Unilabs) at noon on Day 4 after progesterone initiation (after eight doses), one day before FET, to determine adequacy of luteal support. Based on published data prior to inclusion (9, 21), a threshold of 11 ng/ml was predefined as the minimum adequate level. Patients below this threshold received additional subcutaneous progesterone supplementation (Figure 3). No minimal threshold was defined, and all patients underwent embryo transfer. Daily supplementation continued for 12 days until βhCG measurement and was prolonged for eight weeks in cases of positive pregnancy. Progesterone was measured again on Day 12 of supplementation.

### 2.3 Outcome measures

The primary outcome was live birth rate (LBR), defined as the proportions of FETs resulting in delivery of a live-born infant. The objective was to assess the effect of additional subcutaneous progesterone supplementation on LBR among in patients with low baseline levels (<11 ng/ml). Secondary outcomes included biochemical pregnancy rate (BPR), defined as pregnancy diagnosed solely by serum βhCG rise (>10 IU/L and <100 IU/L); clinical pregnancy rate (CPR), defined as presence of at least one gestational sac on ultrasound; and miscarriage rate, defined as spontaneous pregnancy loss before 12 completed weeks of gestation.

### 2.4 Statistical analysis

Continuous outcomes were expressed as mean ± standard deviation, and categorical outcomes as frequencies and percentages. Univariate analysis was performed to describe and compare cycle characteristics and reproductive outcomes between groups. Student's *t*-test was used for continuous variables and Chi-square for categorical variables. Distribution normality was assessed with the Kolmogorov–Smirnov test, showing progesterone distributions were non-Gaussian. Variables were logarithmically transformed to meet parametric assumptions. Rate differences (RD) were calculated using generalized linear models with PLM procedure. Ninety-five percent confidence intervals for differences between proportions were calculated for primary outcomes. All tests were two-tailed, with *p* < 0.05 considered statistically significant. A logistic regression model was used to adjust for confounders using LOGISTIC procedure. Categorical variables were appropriately formatted. Predictor variables were selected based on significance (*p* < 0.2) and literature relevance. Interaction terms were tested to determine whether associations varied by predictor levels. The full model was estimated using maximum likelihood. Interaction significance was assessed with Wald chi-square statistics. Model fit was evaluated with the Hosmer-Lemeshow test (Supplementary Figure). Odds ratios and 95% confidence intervals for main effects and interactions were derived from parameter estimates. Analyses were performed with SAS® version 3.81 (SAS Institute, Cary, NC, USA). A *post hoc* power analysis was conducted to ensure reliability of the results.

## 3 Results

### 3.1 Patient demographics and cycle characteristics

A total of 433 FET cycles met the inclusion criteria. Table 1 summarizes patient demographics and cycle characteristics for all included cycles and compares the Rescue (R-Group) and Control (C-group). The overall mean patient age was 35.8 ± 4.1 years, with a mean BMI of 23.1 ± 3.9 kg/m^2^. No significant differences were observed between groups. Primary infertility was more frequent in the R-Group (75.9%) than in the C-Group (66.2%). Cycle characteristics were similar across groups. Basal anti-Müllerian hormone (AMH) levels averaged 3.9 ± 3.3 ng/ml in the R-Group and 3.9 ± 2.8 ng/ml in the C-Group. Estradiol levels were also comparable (226.4 ± 190.5 pg/ml in the R-Group vs. 224.8 ± 156.7 pg/ml in the C-Group). Endometrial thickness was consistent (8.7 ± 1.3 mm). The mean number of FET cycles per patient was also similar (1.2 ± 0.4 in the R-Group vs. 1.1 ± 0.4 in the C-Group), as was the distribution of Day 5 and Day 6 blastocysts.

**Table 1:** Patients demographical and cycle characteristics.

Characteristics	Overall (*n* = 433)	C-Group (*n* = 235)	R-Group (*n* = 198)	*p*
Age at oocyte retrieval (years)	35.8 ± 4.1	36.1 ± 3.9	35.4 ± 4.3	0.068
Weight (kg)	63.1 ± 12.0	63.3 ± 12.5	62.7 ± 11.4	0.598
BMI (kg/m^2^)	23.1 ± 3.9	23.2 ± 4.0	23.0 ± 3.9	0.727
Primary infertility	70.7%	66.2%	75.9%	0.029
Basal AMH (ng/ml)	3.9 ± 3.1	3.8 ± 3.3	3.9 ± 2.8	0.896
Rank of transfer				
1	55.7%	55.3%	56.1%	0.852
2+	44.3%	44.7%	43.9%	
Estradiol level (pg/ml)^a^	225.6 ± 175.6	226.4 ± 190.5	224.8 ± 156.7	0.930
Endometrial thickness (mm)^a^	8.7 ± 1.3	8.7 ± 1.3	8.7 ± 1.3	0.921
Number of embryos transferred	1.2 ± 0.4	1.2 ± 0.4	1.1 ± 0.4	0.066
Day of blastocyst freezing				
D5	86.8%	87.2%	86.4%	0.790
D6	13.2%	12.8%	13.6%	

The C-Group were patients with adequate serum progesterone levels (≥11.0 ng/ml) on the day before frozen embryo transfer (D4), while the R-Group were patients with low serum progesterone levels (<11.0 ng/ml) on the day before frozen embryo transfer (D4) and received additional daily subcutaneous progesterone injection.

BMI, body mass index; C-Group, control group; R-Group, rescue group.

^a^ Measurements done before progesterone supplementation.

### 3.2 Progesterone levels before and after rescue cycles

**Figure 4:**
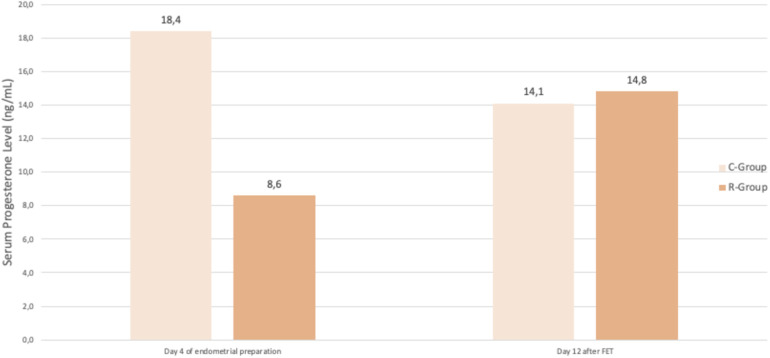
Evolution of progesterone before and after FET. Difference in serum progesterone levels between groups, on Day 4 of endometrial preparation (8.6 ng/ml vs. 18.4 ng/ml; *p* < 0.001) and on day 12 after FET (14.8 ng/ml vs. 14.1 ng/ml; *p* = 0.47), emphasizing the importance of supplementation in restoring adequate progesterone levels. FET, Frozen embryo transfer; C-Group, control group; R-Group, rescue group.

Serum progesterone levels differed between the groups before FET but equalized afterward (Figure 4). On Day 4 of endometrial preparation, serum progesterone was significantly lower in the R-Group, which later received subcutaneous supplementation, than in the C-Group (8.6 ng/ml vs. 18.4 ng/ml; *p* < 0.001). However, at βhCG measurement on Day 12 after embryo transfer, no difference was observed (14.8 ng/ml vs. 14.1 ng/ml; (*p* = 0.47).

### 3.3 Reproductive outcomes

**Figure 5:**
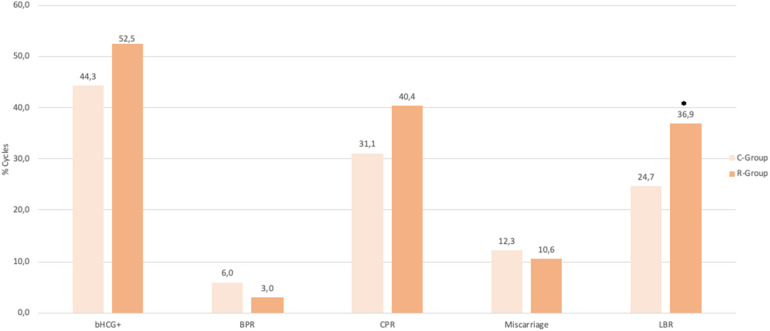
Reproductive outcomes. Reproductive outcomes between C-Group (adequate progesterone levels) and R-Group (low initial levels corrected with subcutaneous supplementation). Pregnancy rates (44.3% vs. 52.5%; *p* = 0.09), BPR (6.0% vs. 3.0%; *p* = 0.15), CPR (31.1% vs. 40.4%; *p* = 0.05), Miscarriage rate (12.3% vs. 10.6%; *p* = 0.57), and LBR (24.7% vs. 36.9%; *p* = 0.006). bhcg+, overall pregnancy rate; BPR, Biochemical pregnancy rate; CPR, Clinical pregnancy rate; LBR, Live Birth rate; C-Group, control group; R-Group, rescue group.

Reproductive outcomes were compared between the C-group (adequate progesterone levels) and the R-group (low initial levels corrected with supplementation) (Figure 5). The overall pregnancy rate was similar: 52.5% (104/198) in R-Group vs. 44.3% (104/235) in C-Group (*p* = 0.09). Biochemical pregnancy rates and clinical pregnancy rates were also comparable. The BPR was 3.0% (6/198) in the R-Group and 5.9% (14/235) in the C-Group, with a rate difference (RD) of −2.9% [95% CI (−6.9; +1.0)]. The CPR was 40.4% (80/198) in the R-Group and 31.1% (73/235) in the C-Group, with an RD of +9.3% [95% CI (−2.0; 20.7)]. The miscarriage rate was 10.6% (21/198) in the R-Group and 12.3% (29/235) in the C-Group, with an RD of −1.7% [95% CI (−8.1; +4.6)]. By contrast, the live birth rate (LBR) was significantly higher in the R-Group at 36.9% (73/198) compared with 24.7% (58/235) in the C-Group, with an RD of +12.2% [95% CI (1.6; 22.7)]. The results of the multivariate regression analysis for variable affecting live birth were presented in Table 2. After adjustment for patient age and basal AMH at oocyte retrieval, type of infertility, BMI, transfer rank, and type and number of blastocyts transferred, the type of luteal support remained an independent predictor of live birth [adjusted OR = 1.79; 95% CI (1.12–2.89)]. *post hoc* power analysis was estimated at 78.6%.

**Table 2:** Logistic regression model to predict live birth.

Effect	Adjusted odds ratio	95% CI (lower)	95% CI (upper)	*p*-Value
**Age at oocyte retrieval (year)**	**0** **.** **88**	**0** **.** **84**	**0** **.** **95**	**<0** **.** **001**
BMI (kg/m^2^)	0.96	0.90	1.02	0.200
Type of infertility (Primary vs. Secondary)	0.89	0.52	1.53	0.677
Basal AMH (ng/ml)	1.03	0.96	1.11	0.433
Rank of transfer (1 vs. 2+)	1.63	1.00	2.66	0.050
**Number of thawed embryo transferred (1 vs. 2)**	**0** **.** **43**	**0** **.** **23**	**0** **.** **79**	**0** **.** **007**
**Type of blastocyst (Day 5 vs. Day 6)**	**2** **.** **35**	**1** **.** **04**	**5** **.** **33**	**0** **.** **041**
**Type of luteal support (R-Group vs. C-Group)**	**1** **.** **79**	**1** **.** **12**	**2** **.** **89**	**0** **.** **016**

Results of a logistic regression model where the outcome variable is live birth and explanatory variables: women's age and basal AMH at oocyte retrieval, number of blastocysts transferred, type of luteal phase support, rank of transfer, type of blastocyst, body mass index (BMI) and type of infertility.

Adjusted odds ratios (OR) are presented along with their 95% Wald confidence intervals (95% CI) and *p* values. Bold values indicate significant independent predictors of live birth.

Model Fit statistics (Hosmer Lemeshow test, *p* = 0.89; pseudo R2 = 0.15; C-statistic = 0.71). Residual analysis showed that deviance and Pearson residuals were approximately centered at zero with standard deviations close to 1, consistent with adequate model fit.

BMI, body mass index; AMH, anti Mullerian Hormone; C-Group, control group; R-Group, rescue group.

## 4 Discussion

This study showed similar pregnancy rates between the compared groups and demonstrated that individualized luteal phase support with additional subcutaneous progesterone could significantly improve live birth rates in HRT-FET cycles. Power analysis indicated that the sample was sufficient to detect the observed effect, although the absolute difference was modest and should be interpreted cautiously given the retrospective design. These findings underscore the importance of personalized progesterone supplementation for patients with low serum progesterone levels (<11 ng/ml) before thawed blastocyst transfer, as this strategy may be beneficial for optimizing outcomes. In HRT-FET, serum progesterone thresholds are critical for reproductive outcomes. A prospective cohort study reported that levels below 8.8 ng/ml (30th percentile of sample distribution) were independently associated with lower ongoing pregnancy rates (22). By contrast, the threshold applied here was closer to the 50th percentile, potentially reflecting poorer treatment adherence. Tailoring luteal support for patients with progesterone values between the 30th and 50th percentiles may have facilitated better reproductive outcomes. Our results are consistent with prior studies showing that patients receiving additional subcutaneous progesterone in HRT-FET cycles achieved better pregnancy outcomes than those treated with vaginal progesterone alone, mainly when baseline levels were below 10.6 ng/ml (18, 19).

Alvarez et al. (19) proposed a tailored approach for patients with low progesterone levels undergoing euploid FET. Their findings indicate that establishing a minimum threshold can enhance reproductive outcomes in HRT-FET with vaginal progesterone. If a low level is identified, it can generally be remedied by administering an additional daily subcutaneous injection. This improvement may result from not only varying administration routes to ensure sufficient exposure but also from optimizing supplementation timing prior to embryo transfer. Labarta et al. (18) found that 25%–30% of women had low midluteal progesterone with 400 mg micronized vaginal progesterone twice daily, leaving one in three to four patients undertreated. These levels were linked to lower ongoing pregnancy. The cause is unclear but may involve poor vaginal absorption. Post-transfer levels were not measured to confirm increases from extra supplementation. Other studies reported improved outcomes with subcutaneous progesterone supplementation (23), though some failed to show benefit (17, 24). For instance, a retrospective cohort analysis investigating rescue protocols including dose doubling for those with serum progesterone <10 ng/ml observed no difference in clinical or ongoing pregnancy rates (25). The present findings highlight the inadequacy of vaginal-only progesterone luteal support in HRT cycles, potentially due to personal conditions like absorption variability, treatment observance, or technical conditions like timing of sampling. Nevertheless, these results support the effectiveness of adding subcutaneous supplementation in stabilizing serum progesterone levels at mid-range values. Implementing stricter thresholds may identify a broader range of patients who could benefit from additional luteal support, ultimately leading to improved implantation and live birth rates. Our study also provides valuable evidence of adequate progesterone levels on Day 12 after FET, supporting early pregnancy maintenance. However, further research is needed to refine progesterone thresholds and clarify their effects across distributions.

### 4.1 Significance of progesterone monitoring

Progesterone induces endometrial secretory transformation, improving receptivity and implantation. While HRT cycles show a relationship between serum progesterone and outcomes, it is unclear whether uterine receptivity depends more on serum or intrauterine levels. A prospective ERA® study showed that endometrial—not serum—progesterone levels directly correlate with receptivity (26). These findings emphasize the importance of uterine-specific progesterone concentration in achieving endometrial receptivity, suggesting that localized progesterone levels are critical in preparing the endometrium for implantation. Monitoring serum progesterone before FET is a cornerstone of effective support. Achieving pre-transfer levels above critical thresholds, such as 10.6 ng/ml, has been consistently associated with improved outcomes (12, 17), while low levels are linked to miscarriage (22). A meta-analysis of 21 cohort studies confirmed that higher midluteal progesterone, specifically above 10 ng/ml, improves ongoing pregnancy and live birth rates, and reduces miscarriage (27). Progesterone administration is as important as monitoring. A double-blind crossover study compared the pharmacokinetics, endometrial histology, transcriptomics, and immune cell composition of oral and vaginal progesterone administration in oocyte donors, founding that, while endometrial immune cell composition differed between treatments, both effectively support endometrial transformation with similar reproductive outcomes (28). However, administration frequency and dose optimization remain important, especially for oral forms, due to faster clearance. Maintaining adequate progesterone levels during the luteal phase is crucial for success. The rescue strategy used here effectively corrected deficiencies in patients with suboptimal baseline levels, and supported implantation and live birth.

### 4.2 Clinical implications

Combining administration routes may optimize both systemic and local uterine progesterone exposure, improving implantation window timing and outcomes in HRT-FET cycles. The estimated number needed to treat for one additional live birth was nine. Variability in progesterone due to pharmacokinetics, BMI, and age suggests that standardized approaches may not be sufficient for all patients and highlights the need for individualized supplementation (12). Subcutaneous progesterone provides systemic exposure comparable to intramuscular administration and has shown equivalent clinical outcomes (29, 30). Furthermore, subcutaneous progesterone brings some notable advantages. The primary benefit is its rapid increase in serum levels —exceeding 14 ng/ml after just two doses of 25 mg daily. It is also better tolerated with fewer side effects such as abdominal discomfort, vaginal bleeding, or an uptick in vaginal discharge (23, 31–33). The convergence of progesterone levels after FET suggests supplementation effectively corrected deficiencies. Previous rescue strategies with oral dydrogesterone, rectal or intramuscular progesterone administrations, or even with doubling micronized vaginal progesterone doses with or without subcutaneous supplementation also showed benefit (25, 29, 34, 35). Such approaches may yield consistent progesterone levels, improving pregnancy outcomes when vaginal-only support is inadequate (23, 33). Further randomized trials are needed to confirm benefits and explore if higher dosing could further improve outcomes for women with low progesterone levels at the time of FET.

### 4.3 Limitations

This study has limitations. The retrospective design may introduce selection bias limiting the generalizability of the findings and precludes causal inference. While consistent with other cohort studies, larger randomized trials are required (30). Another limitation was lack of standardization in timing between last vaginal dose and blood sampling, which may have affected serum progesterone levels despite a narrow window. Another limitation was evaluating only one treatment strategy -subcutaneous progesterone- restricting comparison with alternative interventions (36). Comparing subcutaneous, vaginal, rectal and oral routes of administration could inform more tailored approach to luteal phase support.

### 4.4 Future directions

This study supports subcutaneous rescue supplementation for patients with low progesterone before FET. Findings contribute to the growing evidence that individualized support improves live birth rates and underscore the importance of pre-transfer monitoring. Future work should refine tailored HRT protocols, establish optimal progesterone thresholds by patient characteristics, such as age, BMI, or specific pathologies like endometriosis, and explore pharmacokinetics. Comparative studies of dosage and route combinations of progesterone formulations could optimize personalized luteal support and improve outcomes.

## 5 Conclusion

This study demonstrates the effectiveness of a subcutaneous progesterone rescue protocol, improving live birth rates among women with low pre-transfer progesterone levels in HRT-FET cycles. Individualized luteal support with supplementary subcutaneous progesterone enhances outcomes, offering a practical solution to address insufficient serum levels. Although promising, these results must be interpreted cautiously, as the optimal luteal support protocol in HRT-FET cycles remains unsettled and progesterone regimens remain debated. It is essential to acknowledge the ongoing discussion regarding the most effective method of supplementation for FET cycles. Current treatments validated in fresh transfers are often extrapolated to FET. Further randomized trials with varied strategies are needed to confirm these findings and guide clinical practice.

## Data Availability

The raw data supporting the conclusions of this article will be made available by the authors, without undue reservation.

## Group members of The Ambroise Paré Cherest ART Group

François Xavier Aubriot; Marina Béchard De Spirlet; Véronique Boulard; Jérémy Calvo; Anne Sophie Chevalier; Salah Chouaieb; Stéphane Douard; Estelle Feyereisen; Claire Gauche Cazalis; Vanessa Gayet; Fanta Kanté; Frédéric Lamazou; Yael Lazimy; Joumana Lemaitre; Anne Laure Margulies; Eve Muller; Jad Raad; Patrice Raveneau; Vanessa Ruiz; Valérie Scarabin Carré.

## Supplementary material

Supplementary Figure Regression Model fit statistics using Hosmer-Lemeshow calibration curve. Blue curve = actual calibration. Red dashed line = perfect calibration (y = x line). The model fits the data well (no difference between observed & predicted) *p* = 0.89.

## References

[1] Maheshwari A Bari V Bell JL Bhattacharya S Bhide P Bowler U Transfer of thawed frozen embryo versus fresh embryo to improve the healthy baby rate in women undergoing IVF: the E-freeze RCT. Health Technol Assess. (2022) 26(25):1. 10.3310/AEFU1104 35603917 PMC9376799

[2] Wang J Sauer MV. *In vitro* fertilization (IVF): a review of 3 decades of clinical innovation and technological advancement. Ther Clin Risk Manag. (2006) 2(4):355. 10.2147/tcrm.2006.2.4.355 18360648 PMC1936357

[3] Saket Z Källén K Lundin K Magnusson Å Bergh C. Cumulative live birth rate after IVF: trend over time and the impact of blastocyst culture and vitrification. Hum Reprod Open. (2021) 2021(3):hoab021. 10.1093/hropen/hoab021 34195386 PMC8240131

[4] Lensen S Lantsberg D Gardner DK Sophian AD Wandafiana N Kamath MS. The role of timing in frozen embryo transfer. Fertil Steril. (2022) 118(5):832–8. 10.1016/j.fertnstert.2022.08.009 36150920

[5] Mackens S Santos-Ribeiro S van de Vijver A Racca A Van Landuyt L Tournaye H Frozen embryo transfer: a review on the optimal endometrial preparation and timing. Hum Reprod. (2017) 32(11):2234–42. 10.1093/humrep/dex285 29025055

[6] van de Vijver A Drakopoulos P Polyzos NP Van Landuyt L Mackens S Santos-Ribeiro S Vitrified-warmed blastocyst transfer on the 5th or 7th day of progesterone supplementation in an artificial cycle: a randomised controlled trial. Gynecol Endocrinol. (2017) 33(10):783–6. 10.1080/09513590.2017.1318376 28443690

[7] van de Vijver A Polyzos NP Van Landuyt L Mackens S Stoop D Camus M What is the optimal duration of progesterone administration before transferring a vitrified-warmed cleavage stage embryo? A randomized controlled trial. Hum Reprod. (2016) 31(5):1097–104. 10.1093/humrep/dew045 27005893

[8] Roelens C Santos-Ribeiro S Becu L Mackens S Van Landuyt L Racca A Frozen-warmed blastocyst transfer after 6 or 7 days of progesterone administration: impact on live birth rate in hormone replacement therapy cycles. Fertil Steril. (2020) 114(1):125–32. 10.1016/j.fertnstert.2020.03.017 32553469

[9] Labarta E Mariani G Holtmann N Celada P Remohí J Bosch E. Low serum progesterone on the day of embryo transfer is associated with a diminished ongoing pregnancy rate in oocyte donation cycles after artificial endometrial preparation: a prospective study. Hum Reprod. (2017) 32(12):2437–42. 10.1093/humrep/dex316 29040638

[10] Ghobara T Gelbaya TA Ayeleke RO. Cycle regimens for frozen-thawed embryo transfer. Cochrane Database Syst Rev. (2017) 7(7):CD003414. 10.1002/14651858.CD003414.pub3 28675921 PMC6483463

[11] Mumusoglu S Polat M Ozbek IY Bozdag G Papanikolaou EG Esteves SC Preparation of the endometrium for frozen embryo transfer: a systematic review. Front Endocrinol. (2021) 12:688237. 10.3389/fendo.2021.688237 PMC8299049 34305815

[12] González-Foruria I Gaggiotti-Marre S Álvarez M Martínez F García S Rodríguez I Factors associated with serum progesterone concentrations the day before cryopreserved embryo transfer in artificial cycles. Reprod Biomed Online. (2020) 40(6):797–804. 10.1016/j.rbmo.2020.03.001 32386938

[13] Alsbjerg B Kesmodel US Humaidan P. Endometriosis patients benefit from high serum progesterone in hormone replacement therapy–frozen embryo transfer cycles: a cohort study. Reprod BioMed Online. (2023) 46(1):92–8. 10.1016/j.rbmo.2022.09.005 36216661

[14] Polat M Mumusoglu S Bozdag G Ozbek IY Humaidan P Yarali H. Addition of intramuscular progesterone to vaginal progesterone in hormone replacement therapy in vitrified–warmed blastocyst transfer cycles. Reprod Biomed Online. (2020) 40(6):812–8. 10.1016/j.rbmo.2020.01.031 32362573

[15] Alyasin A Agha-Hosseini M Kabirinasab M Saeidi H Nashtaei MS. Serum progesterone levels greater than 32.5 ng/ml on the day of embryo transfer are associated with lower live birth rate after artificial endometrial preparation: a prospective study. Reprod Biol Endocrinol. (2021) 19(1):24. 10.1186/s12958-021-00703-6 33602270 PMC7890906

[16] Gaggiotti-Marre S Martinez F Coll L Garcia S Álvarez M Parriego M Low serum progesterone the day prior to frozen embryo transfer of euploid embryos is associated with significant reduction in live birth rates. Gynecol Endocrinol. (2019) 35(5):439–42. 10.1080/09513590.2018.1534952 30585507

[17] Cédrin-Durnerin I Isnard T Mahdjoub S Sonigo C Seroka A Comtet M Serum progesterone concentration and live birth rate in frozen-thawed embryo transfers with hormonally prepared endometrium. Reprod Biomed Online. (2019) 38(3):472–80. 10.1016/j.rbmo.2018.11.026 30642638

[18] Labarta E Mariani G Rodríguez-Varela C Bosch E. Individualized luteal phase support normalizes live birth rate in women with low progesterone levels on the day of embryo transfer in artificial endometrial preparation cycles. Fertil Steril. (2022) 117(1):96–103. 10.1016/j.fertnstert.2021.08.040 34548167

[19] Álvarez M Gaggiotti-Marre S Martínez F Coll L García S González-Foruria I Individualised luteal phase support in artificially prepared frozen embryo transfer cycles based on serum progesterone levels: a prospective cohort study. Hum Reprod. (2021) 36(6):1552–60. 10.1093/humrep/deab031 33686413

[20] Gardner DK Sakkas D. Assessment of embryo viability: the ability to select a single embryo for transfer—a review. Placenta. (2003) 24:S5–12. 10.1016/S0143-4004(03)00136-X 14559024

[21] Alsbjerg B Thomsen L Elbaek HO Laursen R Povlsen BB Haahr T Progesterone levels on pregnancy test day after hormone replacement therapy-cryopreserved embryo transfer cycles and related reproductive outcomes. Reprod Biomed Online. (2018) 37(5):641–7. 10.1016/j.rbmo.2018.08.022 30385142

[22] Labarta E Mariani G Paolelli S Rodriguez-Varela C Vidal C Giles J Impact of low serum progesterone levels on the day of embryo transfer on pregnancy outcome: a prospective cohort study in artificial cycles with vaginal progesterone. Hum Reprod. (2021) 36(3):683–92. 10.1093/humrep/deaa322 33340402

[23] Yarali H Polat M Mumusoglu S Ozbek IY Erden M Bozdag G Subcutaneous luteal phase progesterone rescue rectifies ongoing pregnancy rates in hormone replacement therapy vitrified-warmed blastocyst transfer cycles. Reprod Biomed Online. (2021) 43(1):45–51. 10.1016/j.rbmo.2021.04.011 34016521

[24] Brady PC Kaser DJ Ginsburg ES Ashby RK Missmer SA Correia KF Serum progesterone concentration on day of embryo transfer in donor oocyte cycles. J Assist Reprod Genet. (2014) 31(5):569–75. 10.1007/s10815-014-0199-y 24619510 PMC4016380

[25] Arik Alpcetin SI Ince O Akcay B Cevher Akdulum MF Demirdag E Erdem A Comparison of individualized rescue luteal phase support strategies with vaginal and combined vaginal & subcutaneous progesterone administration in artificial frozen-thawed blastocyst embryo transfer cycles based on Serum progesterone levels. Front Endocrinol. (2025) 15:1503008. 10.3389/fendo.2024.1503008 PMC11782913 39897956

[26] Labarta E Sebastian-Leon P Devesa-Peiro A Celada P Vidal C Giles J Analysis of serum and endometrial progesterone in determining endometrial receptivity. Hum Reprod. (2021) 36(11):2861–70 (in Oxford English). 10.1093/humrep/deab184 34382075

[27] Melo P Chung Y Pickering O Price MJ Fishel S Khairy M Serum luteal phase progesterone in women undergoing frozen embryo transfer in assisted conception: a systematic review and meta-analysis. Fertil Steril. (2021) 116(6):1534–56. 10.1016/j.fertnstert.2021.07.002 34384594

[28] Loreti S Thiele K Brucker MD Olsen C Centelles-Lodeiro J Bourgain C Oral dydrogesterone versus micronized vaginal progesterone for luteal phase support: a double-blind crossover study investigating pharmacokinetics and impact on the endometrium. Hum Reprod. (2024) 39(2):403–12. 10.1093/humrep/dead256 38110714

[29] Boynukalin FK Tohma YA Yarkıner Z Gultomruk M Bozdag G Ozkavukcu S Individualized luteal phase support in frozen-thawed embryo transfer after intramuscular progesterone administration might rectify live birth rate. Front Endocrinol. (2024) 15:1412185. 10.3389/fendo.2024.1412185 PMC11239543 39006366

[30] Schütt M Nguyen TD Kalff-Suske M Wagner U Macharey G Ziller V. Subcutaneous progesterone versus vaginal progesterone for luteal phase support in *in vitro* fertilization: a retrospective analysis from daily clinical practice. Clin Exp Reprod Med. (2021) 48(3):262–7. 10.5653/cerm.2020.04021 34370944 PMC8421659

[31] Sator M Radicioni M Cometti B Loprete L Leuratti C Schmidl D Pharmacokinetics and safety profile of a novel progesterone aqueous formulation administered by the s.c. route. Gynecol Endocrinol. (2013) 29(3):205–8. 10.3109/09513590.2012.736560 23127204

[32] de Ziegler D Pirtea D Ayoubi JM. Implantation failures and miscarriages in frozen embryo transfers timed in hormone replacement cycles (HRT): a narrative review. Life. (2021) 11(12):1357. 10.3390/Life11121357 34947887 PMC8708868

[33] Phy JL Weiss WT Weiler CR Damario MA. Hypersensitivity to progesterone-in-oil after *in vitro* fertilization and embryo transfer. Fertil Steril. (2003) 80(5):1272–5. 10.1016/S0015-0282(03)01170-1 14607588

[34] Vidal A Dhakal C Werth N Weiss JM Lehnick D Schwartz K Supplementary dydrogesterone is beneficial as luteal phase support in artificial frozen-thawed embryo transfer cycles compared to micronized progesterone alone. Front Endocrinol. (2023) 14:1128564. 10.3389/fendo.2023.1128564 PMC10042263 36992810

[35] Alsbjerg B Jensen MB Povlsen BB Elbaek HO Laursen RJ Kesmodel US Rectal progesterone administration secures a high ongoing pregnancy rate in a personalized hormone replacement therapy frozen embryo transfer (HRT-FET) protocol: a prospective interventional study. Hum Reprod. (2023) 38(11):2221–9. 10.1093/humrep/dead185 37759346 PMC10628493

[36] Child T Leonard SA Evans JS Lass A. Systematic review of the clinical efficacy of vaginal progesterone for luteal phase support in assisted reproductive technology cycles. Reprod Biomed Online. (2018) 36(6):630–45. 10.1016/j.rbmo.2018.02.001 29550390

