# Supplementary figures and images for: Effect of a luteal phase rescue protocol on live birth rates in frozen embryo transfer cycles
Source: Front Reprod Health. 2025 Sep 30;7:1547939. doi: 10.3389/frph.2025.1547939 (PMC12518300; doi:10.3389/frph.2025.1547939)

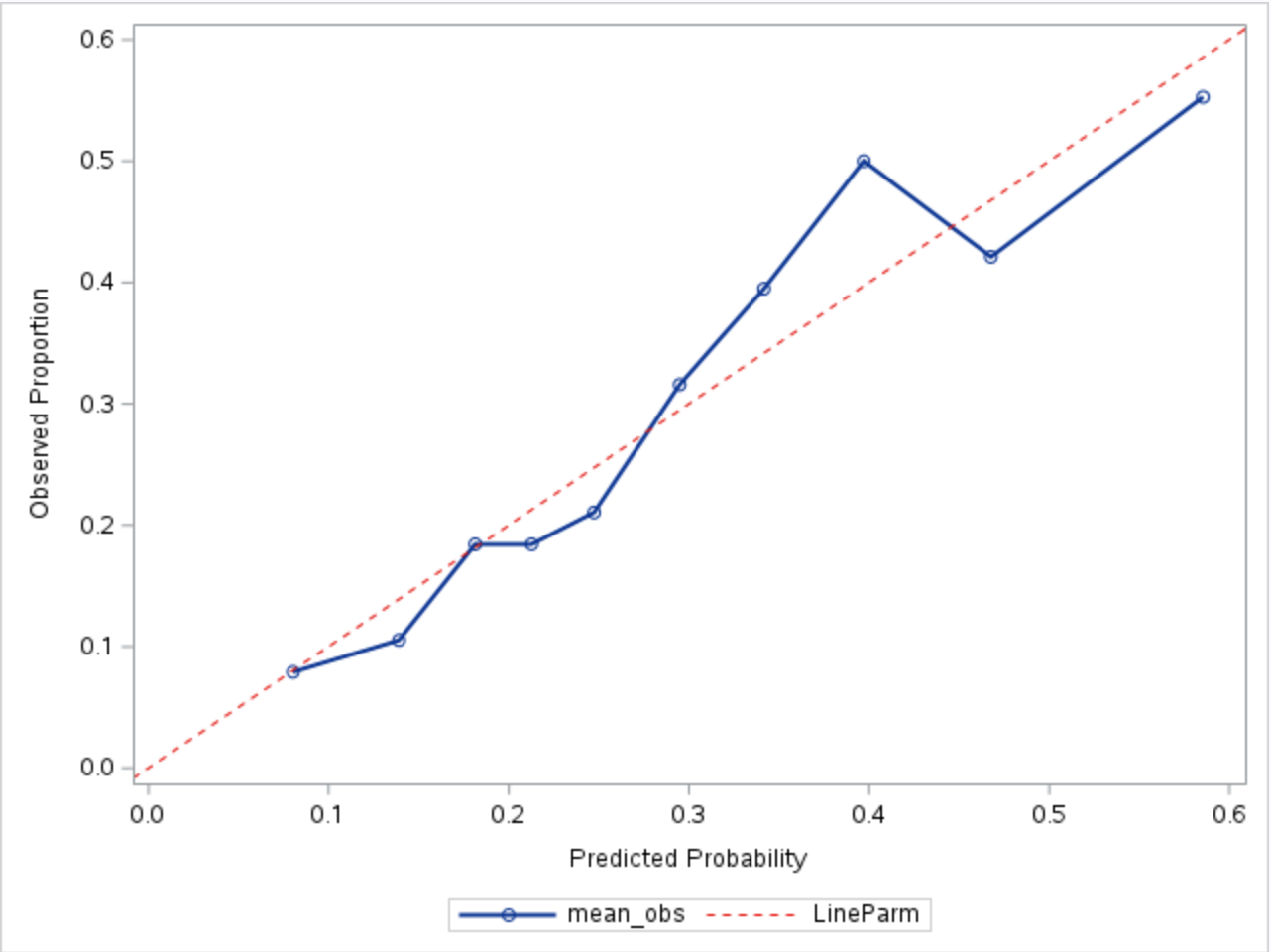

Supplement: Supplementary Figure — Regression Model fit statistics using Hosmer-Lemeshow calibration curve. Blue curve = actual calibration. Red dashed line = perfect calibration (y = x line). The model fits the data well (no difference between observed & predicted) p = 0.89. [file Image1.jpeg]
